# Food IgG_4_ antibodies are elevated not only in children with wheat allergy but also in children with gastrointestinal diseases

**DOI:** 10.1186/s12876-016-0450-3

**Published:** 2016-03-22

**Authors:** Grażyna Czaja-Bulsa, Michał Bulsa, Aneta Gębala

**Affiliations:** Pediatrics and Pediatric Nursery Unit of The Pomeranian Medical University in Szczecin, ul. Żołnierska 48, 71-210 Szczecin, Poland; Division of Paediatrics, Gastroenterology and Rheumatology of The „Zdroje” Hospital in Szczecin, Szczecin, Poland; Independent Laboratory of Propaedeutics in Paediatrics of The Pomeranian Medical University in Szczecin, Szczecin, Poland

**Keywords:** Children, Coeliac disease, Helicobacter pylori infection, Specific IgG, Specific IgG_4_, Wheat allergy

## Abstract

**Background:**

Food sIgG and sIgG_4_ are highly individually versatile. We put a hypothesis that one of the responsible factors is the presence of gastrointestinal inflammatory diseases. The objectives were: 1. An analysis of wheat and rice sIgG and sIgG_4_ in healthy children, children with IgE-mediated wheat allergy (WA), coeliac disease (CD) and Helicobacter pylori infection (HP). 2. Usability of wheat sIgG and sIgG_4_ in the WA diagnostics.

**Methods:**

We compared 388 each wheat and rice sIgG and sIgG_4_ in a group of 200 children: 50 WA (diagnosis, diet treatment, tolerance), 50 CD (diagnosis and remission), 50 HP and 50 healthy. SIgE, sIgG, sIgG_4_ were determined with the FEIA method (Pharmacia CAP System).

**Results:**

In healthy children food sIgG were the lowest; no sIgG_4_ were found. In the CD diagnosis group wheat and rice sIgG and rice sIgG_4_ were the most common and their concentrations were the highest (*p* < .001, *p* < .05). Wheat sIgG_4_ were the highest in WA children (diagnosis and tolerance) to fall during the elimination diet (*p* < .05). Wheat and rice sIgG remained the same in all allergy phases. Rice sIgG also did not differ in the class G_4_.

**Conclusions:**

1. Serum concentrations of wheat and rice sIgG and sIgG_4_ are elevated in children with CD, HP and WA. 2. Sub-clinical incidence of some gastrointestinal inflammatory diseases may be responsible for high individual versatility of food sIgG and sIgG_4_ concentrations in serum. 3. Wheat sIgG and sIgG_4_ in children do not correlate with WA clinical picture.

## Background

Along with the recent development of diagnostic techniques it has become increasingly popular to use serum concentration levels of specific IgG (sIgG) and IgG_4_ (sIgG_4_) as markers of food hypersensitivity. Vast popularity of these diagnostically incorrect tests provoked EAACI to issue an official statement which was later supported by AAAAI and CSACI [1–3]. Scientific associations do not recommend using sIgG and sIgG_4_ assays in the food hypersensitivity diagnostics. They point out that many individuals have their elevated levels which do not correspond to clinical symptoms of the disease. The research has shown that in humans the presence of food sIgG and sIgG_4_ is highly individual. The sIgG appear in half of the population, usually as a response to the most common foods [4]. The sIgG_4_ is only in the case of some food allergens of cow’s milk and egg protein. It has not been resolved so far why in healthy people the frequency and the titers of food sIgG and sIgG_4_ show such substantial individual variations. Does it depend merely on the frequency of food consumption and the nature of the antigen? Or are there any additional factors?

In this paper we make a hypothesis that these factors can be gastrointestinal inflammatory diseases. One of them is coeliac disease (CD) which can be asymptomatic [5]. The adults with untreated CD showed higher sIgG activity for gliadin, casein and ovalbumin [6, 7]. There have been no research in this respect into other gastrointestinal diseases, hence the question arises if in their case the titers of food sIgG and sIgG_4_ are different from normal. Some of these diseases, e.g. the infection with Helicobacter pylori (HP), are very common and can take a non-symptomatic or mildly symptomatic form or its symptoms may be non-specific.

The purpose of this paper is a comparative analysis of the frequency and titers of wheat and rice sIgG and sIgG_4_ in healthy children and the children with IgE-mediated wheat allergy (WA), with CD and HP. Moreover, we evaluate the usefulness of assays of wheat sIgG and sIgG_4_ in the WA diagnostics. Although wheat is one of the most common food allergens in children, the presence of food sIgG and sIgG_4_ in WA has’nt been discussed in the literature.

## Methods

We compared 338 assays each of wheat and rice sIgG and sIgG_4_ antibodies determined in 200 children in four groups: 50 children with WA (50 assays each at the time of diagnosis and during the elimination diet; 38 assays during the tolerance); 50 children with CD (diagnosis – aCD, remission - rCD), 50 children with HP and 50 children from the control group (Table 1). Information was collected about the subjects’ consumption of wheat and rice (a questionnaire). The course of WA in a group of 50 children described in this paper (clinical picture over the years, tolerance development age and its factors, specific IgE and IgE on diagnosis, during diet treatment and tolerance) were specified earlier in References #8.

**Table 1:** Characteristics of the study patients

Study groups	No.	Sex	Age (mo.)	
		Male	Median	Range
Control group	50	26	66,4	12 – 197
Wheat allergy				
-Diagnosis -Diet treatment -Tolerance	50 50 38	32 32 20	13,0 36,0 69,5	2 – 22 21 – 42 37 – 192
Coeliac disease				
-Active -Remission	50 50	19 19	76,3 98,6	33 – 230 57 – 254
Helicobacter pylori infection	50	23	97,4	58 – 212

WA was diagnosed in children with positive food challenge results (double-blind placebo-controlled food challenge, DBPCFC) with symptoms occurring within 2 h after wheat consumption and positive SPT as well as with the levels of wheat sIgE higher than 0.7 kU/L. The first challenge test was performed as the open food challenge (OFC), the second - always as the DBPCFC followed by the OFCs. DBPCFC has been described in detail before [8]. 38 children developed tolerance. The subjects were diagnosed with other food allergies, most frequently to cow’s milk, egg protein and peanuts. During the observation the majority of children were diagnosed with atopic dermatitis (78 %), asthma (48 %) and allergic rhinitis (34 %). The progress of WA has been described in detail in References #8. The sIgG and sIgG_4_ assays were made at the time of diagnosis (the median age was 13 months: 2–22 months.), after 2 years of treatment with a wheat-free diet (the median age was 36 months: 21–42 months.) and during the period of tolerance - negative OFC with wheat (the median age was 69.5 months: 37–192 months).

CD children were examined at the time of diagnosis – aCD (76.3 months: 33 – 230 month.) and remission – rCD (98.6 months: 57 – 254 months). The aCD was made for children with the malabsorption syndrome (Marsh IIIC) and with antibodies to tissue transglutaminase as well as with endomysial antibodies [5]. The rCD was diagnosed when this antibodies were normal. The sIgG and sIgG_4_ assays were not made until after 12 monthsnths of remission, usually 2 years after the diagnosis.

HP was diagnosed in children (97.4 months: 58 – 212 mo.) with typical endoscopic (gastritis, positive CLO test) and histological picture (chronic gastritis) [9].

The control group (66.4 months: 12 – 197 months.) was composed of patients in whom lesions in the upper part of the digestive tract were ruled out (endoscopic and histological examination). Children had been admitted due to abdominal pains or dwarfism (suspected malabsorption syndrome). They were diagnosed with functional gastrointestinal disorders or constitutional dwarfism.

The inclusion criteria were: diagnosed WA, CD or HP infection and the consent of participants and their guardians.

The criteria for a child to be excluded from observation were other chronic diseases and decreased concentration of total IgG. For children from the CD group, the HP group and the control group an additional elimination factor was atopy which was diagnosed on the basis of an elevated level of tIgE and/or positive sIgE test results for 8 allergens (milk, egg, peanut, cat, Cladosporium herbarum, Dermathophagoides pteronyssinus, grass, sagebrush) and/or a positive SPT (10 air-borne and 10 food allergens). 56 children were lost to follow-up: 52 children with atopy and from WA group: 1 child with ulcerative colitis and 3 children who visited the clinic only once.

All the patients remained under the care of an attending physician (an allergologist or gastroenterologist) throughout the whole observation period. Children were patients of the outpatient clinic and the gastroenterology department. The study was conducted from January 1990 to May 2012.

The titers of tIgE (accuracy 2–9,1 %, sensitivity <2 kU/L, specificity 100 %), sIgE (accuracy 5–11 %, sensitivity <0,35 kU/L, specificity 100 %), sIgG (accuracy 5–10 %, sensitivity <20ug/L, specificity 100 %) and sIgG_4_ (accuracy 4–7 %, sensitivity <1,5ug/L, specificity >95 %) to wheat and rice were determined by means the FEIA method using the ImmunoCAP System (UniCAP, CAP 100; Pharmacia & Upjohn Diagnostics AB, Uppsala, Sweden). SPTs were performed by means of commercial solutions manufactured by Allergopharma (Germany). The wheal reactions were read after 15 min. (histamine) - 20 min (allergens). Tests were considered positive when the wheal size was ≥ 3 mm in diameter in comparison with the negative control (control solution).

The data were characterized by the median, the minimum and maximum values. The Mann–Whitney test was used to compare sIgG and sIgG_4_ levels among the patients in the examined groups. The correlation between the selected variables was evaluated with the Spearman rank correlation. A tool used for the statistical analysis was STATA 11, License No 30110532736.

The study was conducted in compliance with the Declaration of Helsinki and following the consent by the Ethical Committee of the Pomeranian Medical University in Szczecin (BN-001/107/90). Sources of funding for the research: State Committee for Scientific Research grant No4 PO5E 086 14 and the clinic in-house research budget.

## Results

The children from the control group as well as the ones with WA (diagnosis and tolerance), aCD and HP consumed wheat daily and rice – 1–2 times a week. The children in rCD and the WA children did not consume wheat, but they were given rice at least 4 times a week.

### Wheat and rice sIgG_4_ are not present in children from the control group

**Fig. 1:**
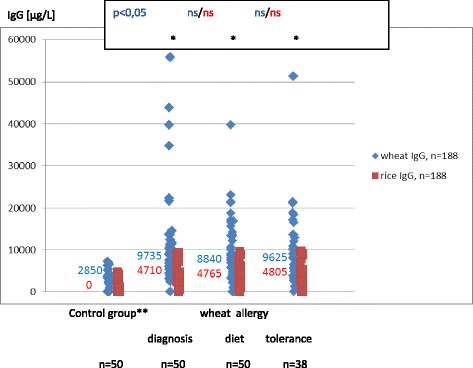
Serum concentrations and medians of wheat and rice sIgG in children from the control group and in children with IgE mediated wheat allergy. *wheat/rice: *p* < 0,05; Mann–Whitney test, **control group: children not suffering from atopy and gastrointestinal diseases in the upper part of the digestive tract (endoscopic and histological examination). In the control group the median titer of wheat sIgG was 2850 μg/L (0–7250 μg/L), while that of rice sIgG was 0 (0–4780 μg/L). In each of the wheat allergy phases the titers of sIgG were significantly higher for wheat than for rice (*p* < .005). They did not differ significantly over the three allergy phases both in the case of wheat and rice

In the children from the control group wheat sIgG was found in 66 % of subjects and rice sIgG – in 30 % (Table 2). No wheat or rice sIgG_4_ was found in any of those children. The median titer of wheat sIgG was 2850 μg/L (0–7250 μg/L), while that of rice sIgG was 0 (0–4780 μg/L) (Fig. 1).

**Table 2:** Incidence of wheat and rice sIgG and sIgG_4_ antibodies in children from the study groups

Study groups	Wheat IgG (%)	Wheat IgG_4_ (%)	Rice IgG (%)	Rice IgG_4_ (%)
Control group	66	0	30	0
Wheat allergy				
-Diagnosis -Diet treatment -Tolerance	96 98 98	92 88 94	94 92 94	50 46 48
Coeliac disease				
-Active -Remission	100 84	86 54	100 84	50 16
Helicobacter pylori infection	78	76	56	12

### Wheat and rice sIgG and IgG_4_ are elevated in children over the three wheat allergy phases

**Fig. 2:**
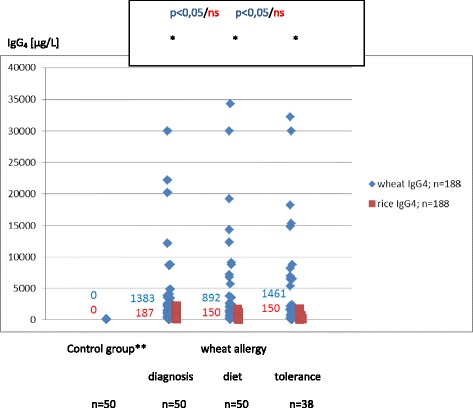
Serum concentrations and medians of wheat and rice sIgG4 in children from the control group and in children with IgE mediated wheat allergy. *wheat/rice: *p* < 0,05; Mann–Whitney test, **control group: children not suffering from atopy and gastrointestinal diseases in the upper part of the digestive tract (endoscopic and histological examination). In children from the control group wheat or rice sIgG_4_ were not found. In each of the wheat allergy phases the titers of sIgG_4_ were significantly higher for wheat than for rice (*p* < .001). Wheat sIgG_4_ titers were the same at the time of diagnosis and in the period of tolerance, and decreased during the wheat-free diet (*p* < .05). Rice sIgG_4_ the titers were also the same over the three allergy phases

At the time of WA diagnosis, diet treatment and WA tolerance, wheat sIgG and sIgG_4_ and rice sIgG were found in the majority of subjects (88–96 %), while rice sIgG_4_ – in 46–50 % (Table 2). In each of the WA phases the titers of sIgG and sIgG_4_ were significantly higher for wheat than for rice (*p* < .005 and *p* < .001) (Figs. 1, 2). In the class G they did not differ significantly over the three allergy phases both in the case of wheat and rice (Fig. 1). Rice sIgG_4_ the titers were also the same over the three allergy phases (Fig. 2). Wheat sIgG_4_ titers were the same at the time of diagnosis and in the period of tolerance, and decreased during the wheat-free diet (*p* < .05).

### Wheat and rice sIgG and IgG_4_ are elevated in children with coeliac disease and Helicobacter pylori infection

**Fig. 3:**
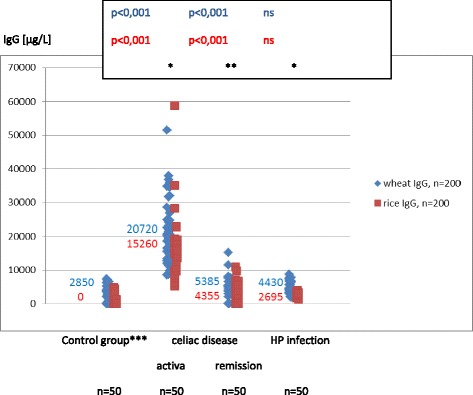
Serum concentrations and medians of wheat and rice sIgG in children from the control group and in children with coeliac diseases and Helicobacter pylori infection. wheat/rice: *p < 0,05, **ns; Mann–Whitney test, ***control group: children not suffering from atopy and gastrointestinal diseases in the upper part of the digestive tract (endoscopic and histological examination). The titers of wheat and rice sIgG were always more elevated in children with active celiac disease than in remission of celiac disease (*p* < .001). In active celiac disease the serum concentrations of wheat sIgG were higher than the rice-specific ones (*p* < .05), to remain the same in remission of celiac disease. In the children with Helicobacter infection the median wheat sIgG were higher than that for rice (*p* < .05). Their serum concentrations of wheat sIgG were the same as in children with the remission of celiac disease

**Fig. 4:**
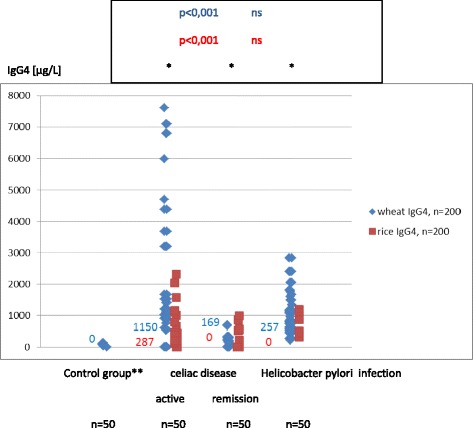
Serum concentrations and medians of wheat and rice sIgG_4_ in children from the control group and in children with coeliac diseases and Helicobacter pylori infection. *wheat/rice: *p* < 0,05; Mann–Whitney test, **control group: children not suffering from atopy and gastrointestinal diseases in the upper part of the digestive tract (endoscopic and histological examination). The titers of wheat and rice sIgG_4_ were always more elevated in children with active celiac disease than in children with remission of celiac disease (*p* < .001). In children with active celiac disease the serum concentrations of wheat sIgG_4_ were higher than the rice-specific ones (*p* < .05), to remain the same in children with remission of celiac disease. In the children with the Helicobacter pylori infection the median wheat of sIgG_4_ were higher than that for rice (*p* < .05). Their serum concentrations of wheat sIgG_4_ were the same as in children with the remission of celiac disease

In the period of aCD wheat and rice sIgG were present in all the subjects, sIgG_4_ were less common (86 % and 50 %) (Table 2). In the rCD the antibodies were rarer: wheat and rice IgG was found in 84 % of subjects and sIgG4 - in 54 % and 16 %. The titers of wheat and rice sIgG and sIgG_4_ were always more elevated in aCD than in rCD (*p* < .001) (Figs. 3, 4). In aCD the serum concentrations of wheat sIgG and sIgG_4_ were higher than the rice-specific ones (*p* < .05), to remain the same in rCD.

In the HP children wheat sIgG and sIgG_4_ were found in 78 % and 76 % respectively, while rice sIgG and sIgG_4_ were less common (56 % and 12 %). The median wheat sIgG and sIgG_4_ were higher than that for rice (*p* < .05) (Figs. 3, 4).

In children in aCD wheat and rice sIgG and rice sIgG_4_ were the most elevated (*p* < .001, *p* < .001 and *p* < .05) (Figs. 3, 4). In the sIgG_4_ subclass the highest titers for wheat were observed in WA children in the phase of diagnosis and tolerance, while in the period of wheat-free diet they were identical as in aCD (Fig. 2).

## Discussion

In our study we examined the frequency and titers of wheat and rice IgG and IgG_4_. We compared a group children not suffering from atopy and gastrointestinal diseases in the upper part of the digestive tract (control group) to a group of children with WA and with CD and HP, two common and usually mildly symptomatic gastrointestinal diseases. Atopy had been excluded from the study since it is known to predispose to developing food sIgG and sIgG_4_ [7, 10, 11].

### Wheat and rice sIgG are not present in all healthy children (control group) but are more common in children with wheat allergy, coeliac disease and Helicobacter pylori infection

The results confirm other authors’ observation that food sIgG do not develop in all healthy children. In addition to that, their incidence and titers vary depending on the food [4]. In this study we detected double as much wheat sIgG as rice IgG (66 % and 30 %) (Table 2). Our research has also shown that wheat sIgG reach higher titers than the ones that are rice specific. In children with WA, CD and HP not only did we observe more common wheat and rice sIgG, but they also reached higher concentration levels in serum (Figs. 1, 3). We did not find the wheat and rice sIgG_4_ fraction in children from the control group who suffered neither from atopy nor from any gastrointestinal disease (Table 2). The fraction was present in children from the remaining groups: WA, CD and HP (Figs. 2, 4).

The highest concentrations and frequency of wheat and rice sIgG were observed in aCD, while in rCD their frequency was lower and their titer values were smaller (Fig. 2).

### Wheat and rice IgG_4_ don’t present in healthy children (control group) but are more common in children with wheat allergy, coeliac disease and Helicobacter pylori infection (Table 2)

The results confirm other authors’ observation that food IgG_4_ do not develop in healthy children as a reaction to all kinds of food. As a prevailing subclass they are synthesized for some food allergens only, mainly to chicken egg protein and cow’s milk. They are most common and reach the highest titers for the chicken egg protein ovomucoid, while in the case of cow’s milk casein, gliadin and gluten the system first of all synthesizes subclasses 1 and 3 of immunoglobulin G [6].

### Wheat sIgG and sIgG_4_ do not correlate with the wheat allergy clinical picture

In the majority of children with WA and CD the humoral response to wheat in the class sIgG and sIgG_4_ was persistent – specific antibodies were present in serum even after wheat had been eliminated from the diet. In WA they were still found in most of the subjects, sIgG titer did not change and sIgG_4_ decreased (*p* < .05). In rCD their concentration fell considerably. Persisting long-term highly stable sIgE and sIgG_4_ epitope-binding patterns were also observed among patients with peanut allergy who were avoiding the allergen [12, 13].

In contrast to children with WA, the elimination of a food from healthy children’s diet results in decreased sIgG titers [14]. Also in milk allergic small children being on a milk-free diet a considerable fall in sIgG serum concentration was observed, which had taken place before their tolerance to milk was developed [15, 16]. Infants with longstanding presence of milk or hen’s egg sIgG suffered from a persistent allergy to those foods [17].

We also found out that the frequency and titers of wheat sIgG and sIgG_4_ did not correlate to the WA clinical picture. They were developed by the majority of children, but not in all of them, and their frequency was the same in all the disease phases (Table 2). Rice sIgG was observed in the majority of children in all the WA phases, while rice sIgG_4_ - in a half of the subjects. This indicates that in WA the immune response is changed. It refers not only to food allergens, but also to tolerated foods.

However, several opposite findings have been published that the sIgG and sIgG_4_ titers do not differ between healthy individuals and patients with atopy or with food allergy to cow’s milk, chicken’s egg and kiwi [16, 18, 19]. Tay et al. found that serum concentration of ovalbumin sIgG and sIgG_4_ is identical in healthy, allergic and tolerant individuals, but in the case of peanut sIgG and sIgG_4_ the titers are higher in allergic and atopic patients than in healthy subjects [10].

It is generally assumed that in children with food allergy the elevated titers of food sIgG are the effect of their increased intestinal permeability. When an allergen is eliminated from a diet, intestinal permeability normalizes [20]. In our study group, most of the WA children (78 %) suffered from atopic dermatitis as well. As Majamaa and Isolauri have found out, in such patients the transport of food macromolecules is seven times larger than in healthy individuals and it includes 20 % of the absorbed proteins [21, 22].

The development of food tolerance, both natural and in oral immunotherapy, is associated with the increase in food sIgG_4_ [23–26]. The maintenance of tolerance to cow’s milk in atopic individuals is characterized by the fall in sIgE titers and high levels of sIgG_4_ [27]. Children with egg allergy who have developed tolerance to baked egg also experience lowered sIgE and elevated sIgG_4_ concentrations [28]. Low titers of sIgG_4_ in young children with food allergy indicate its longstanding character. High serum concentrations of sIgG_4_ is characteristic to these food allergic children who develop their tolerance early [29, 30]. Sletten et al. claimed opposite findings that in children with milk allergy high titers of sIgG_4_ for milk allergens decrease during the tolerance period [31].

In conclusion, the incidence and titers of sIgG and sIgG_4_ are determined not only by the type of food, but also by the type and the activity of a gastrointestinal disease. Higher incidence and titers of food sIgG and sIgG_4_ were observed in all the diseases under study, i.e. in WA, CD and HP. This indicates that gastrointestinal diseases, due to its commonness and mildly-symptomatic nature, can be one of the factors responsible for high individual versatility of food sIgG and sIgG_4_.

### The highest concentration of wheat and rice sIgG are in children with coeliac disease

High concentrations of food sIgG in serum are characteristic of CD (Fig. 3). They are elevated also at the time of remission when they reach slightly higher titers than in HP infection. Elevated sIgG_4_ is not a distinctive feature of CD as it is secondary to the rise in sIgG. Still, in CD these antibodies reach top values that are just slightly lower than in WA where the wheat sIgG_4_ synthesis is preferential (Figs. 2, 4). What is more, it is worth noting that in WA and CD the enhanced synthesis of sIgG and sIgG_4_ includes not only the symptom-inducing food (wheat), but also the food which is tolerated (rice). This confirms that in WA, CD and HP the immune response to various foods is changed.

### Wheat sIgG and sIgG_4_ are useless in the wheat allergy diagnostics

The findings of our study also point to the fact that the assays of wheat sIgG and sIgG_4_ are useless in the WA diagnostics. In WA the concentration of these antibodies increases as a response to not only wheat, but to other foods as well, hence it is impossible to determine the actual allergen. The elevated titers persist throughout all the disease phases, so patients recently diagnosed for allergy cannot be distinguished from those who are being treated with elimination diet or have entered the period of tolerance.

The above findings are burdened by a small size of the sample (50 children in each of the groups). Therefore, it is necessary to confirm the findings in a larger group of patients.

## Conclusions

1. Serum concentrations of wheat and rice sIgG and sIgG_4_ are elevated in children with coeliac disease, Helicobacter pylori infection and IgE-mediated wheat allergy.
2. Sub-clinical incidence of some gastrointestinal inflammatory diseases may be responsible for high individual versatility of food sIgG and sIgG_4_ concentrations in serum.
3. Wheat sIgG and sIgG_4_ in children do not correlate with IgE-mediated wheat allergy clinical picture.

## Abbreviations

AAAAI: American Academy of Allergy, Asthma & Immunology
aCD: coeliac disease diagnosis
CD: coeliac disease
CLO test: test for Helicobacter pylori diagnosis in tissue
CSACI: Canadian Society of Allergy and Clinical Immunology
DBPCFC: double-blind placebo-controlled food challenge
EAACI: The European Academy of Allergy and Clinical Immunology Organisation
FEIA method: fluoro-immuno-enzymatic method
HP: helicobacter pylori infection
M: male
mo.: months
OFC: open food challenge
rCD: remission of coeliac disease
sIgE: specific immunoglobulin E
sIgG: specific immunoglobulin G
sIgG4: specific immunoglobulin G4
SPT: skin prick test
tIgE: total immunoglobulin E
WA: IgE-mediated wheat allergy
